# A Closed Reduction of Cervical Spine Subluxation in an Arabian Foal with an External Neck Stabilizer

**DOI:** 10.3390/ani15030325

**Published:** 2025-01-23

**Authors:** Natalia Domańska-Kruppa, Elżbieta Stefanik, Małgorzata Wierzbicka, André Kleinpeter

**Affiliations:** 1 Department of Large Animal Diseases and Clinic, Institute of Veterinary Medicine, Warsaw University of Life Sciences, Nowoursynowska 100, 02-797 Warsaw, Poland; elzbieta_stefanik@sggw.edu.pl (E.S.); malgorzata_wierzbicka@sggw.edu.pl (M.W.); 2 Equi Centrum Karkosy, Karkosy 18A, 99-100 Łęczyca, Poland; 3 Tierklinik Alt Sammit, Lerchenweg 1a, 18292 Alt Sammit, Germany; andre.kleinpeter@gmail.com

**Keywords:** cervical spine subluxation, foal, neck injury, closed reduction in cervical subluxation

## Abstract

Cervical spine injuries that impact young horses and foals can result in mild to severe neurological signs or even result in sudden death. There are only a few reports on conservative treatment options for this condition in the scientific literature. If the condition is left untreated, it can lead to the development of degenerative joint disease, resulting in chronic neurological symptoms and discomfort. We present the case of a two-day-old Arabian foal that showed signs of ataxia following a neck injury, being the result of cervical spine subluxation. Radiological examination revealed a dislocation between the second and third cervical vertebrae. At admission to the clinic on the seventh day of life, the foal’s clinical examination parameters were within physiological ranges. The head posture at the presentation was consistently low, the foal could not lift its head above the shoulder joint throughout the whole examination, the neck muscles were spastically tensed and clinical signs of ataxia were present. The foal underwent a closed reduction in the subluxation under general anesthesia and a fiberglass semicircular gutter was created to stabilize the neck in the desired position. The ataxia symptoms began to improve around day 12 post manipulation, and the fiberglass stabilizer was removed after 16 days post manipulation, followed by radiographs. The dislocation of C2/C3 was no longer visible on the radiographs, and the foal was able to assume a normal neck posture after the removal of the fixator.

## Simple Summary

Foals and young horses that sustain cervical spine injuries as a result of high-speed accidents are prone to developing neurological signs that range from mild to severe or even result in sudden death. This case report describes a successful nonsurgical reduction of subluxation between the second and third cervical vertebrae in a foal and its short-term outcome. The foal was discharged home without any neurological deficits after treatment.

## 1. Introduction

Cervical spine injuries that cause spinal cord injury in young horses and foals can cause neurological signs or even sudden death [1,2]. These injuries include subluxations or luxations, fractures of the cervical vertebrae and separation of the growth plates, and sometimes there is no overt bony injury at all [3]. Subluxation is a condition in which the joint surfaces are displaced relative to each other without completely losing contact with each other [4]. Subluxation can be congenital or acquired. Subluxation of the atlantoaxial joint has been described as being a result of a hereditary malformation of the occipitoatlantoaxial joints in Arabian foals. Acquired incomplete dislocations of the cervical spine are rare and are most often the consequence of trauma [5].

## 2. Materials and Methods

### Case Presentation

**Figure 1:**
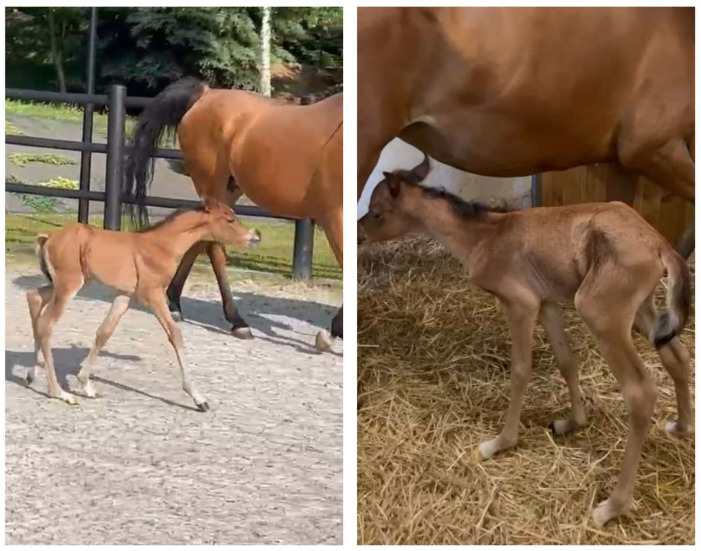
Photos taken 4 days after the injury—the foal presented a posture with a stiff and characteristically hyperextended neck as well as clinical signs of ataxia.

A two-day-old Arabian foal sustained a severe neck injury after its dam was startled in the stall and ran into the foal. The foal subsequently fell and rolled multiple times while trying to stand back up. Immediately after the accident, the owner described disorientation and an unsteady gait, but the foal was able to nurse on its own. The following day, the owner described swelling on the right side of the neck and pain when touching the swollen area. Because the foal could suckle on its own, a decision was made to observe it temporarily. On the fourth day after the injury, the clinical signs of ataxia became significantly more severe and the neck assumed an abnormal, stiff hyperextended position with the head stretched forward (Figure 1).

**Figure 2:**
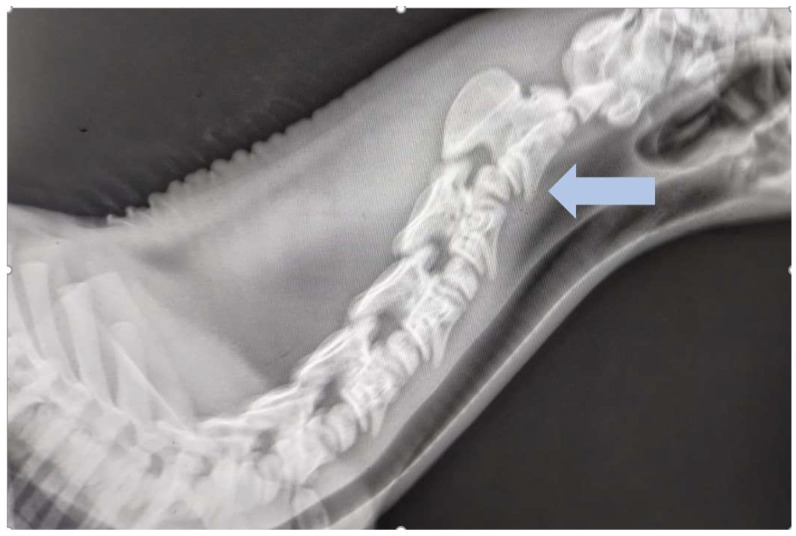
Latero-lateral view of the first to sixth cervical vertebrae of an ataxic foal with severe neck stiffness and pain. There is subluxation of the second and third cervical vertebrae (blue arrow). Courtesy of Equinus, DVM Izabela Pikuła.

The referring veterinarian performed a radiological examination in the stable. Latero-lateral and dorsoventral oblique views (Figure 2) of the neck were obtained. The latero-lateral view showed significant subluxation between C2 and C3 and surgical consultation was recommended.

The owners took the foal to the equine clinic on the seventh day of life. At admission, the foal’s clinical examination parameters were within physiological ranges and the foal was calm and attentive during the examination. The heart rate was 66 beats/min, the respiratory rate was 36 breaths/min, the body temperature was 38.1 °C, and the mucous membranes were pink. Blood tests (cell blood count and biochemistry) did not reveal any abnormalities. Significant proprioceptive ataxia of the fore and hindlimbs were evident during the walk, especially in small circles. The degree of ataxia was graded as 2 out of 6 according to the Modified Mayhew Ataxia Scale [1]. Regardless of the direction of the circle, the severity of ataxia remained the same. During the trot, the foal’s gait was very stiff and attempts to move to a faster gait were unsuccessful. During the whole examination, the foal could not lift its head above the shoulder joint and the neck muscles were tensed. No pathological changes were observed in cranial nerves.

Neck tenderness was slightly increased on both sides; on the right side at the C2/C3 level, painful enlargement of soft tissues was palpable. Based on the examination results, a decision was made to administer anti-inflammatory and analgesic treatment. Meloxicam (Rheumocam 15 mg/mL, Chanelle Pharmaceuticals Manufacturing Ltd., Galway, Republic of Ireland) was administered orally at a dose of 0.6 mg/kg b.w. for 14 days, and dimethyl sulfoxide (DMSO) was administered intravenously three times every 48 h at 1 g/kg b.w.

**Figure 3:**
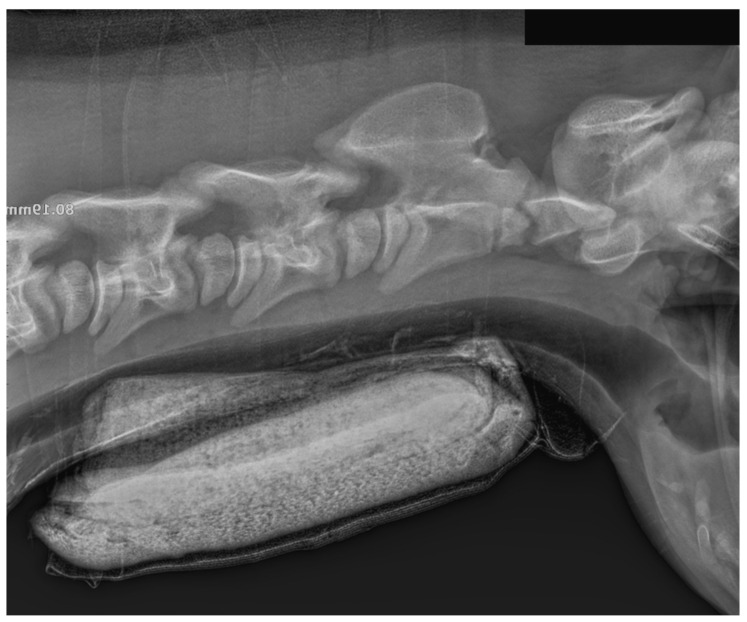
Radiograph of the neck in the latero-lateral projection. The radiograph was taken immediately after manual repositioning of the neck and stabilization with a semicircular fiberglass gutter on the ventral surface of the neck. Visible cervical vertebrae are in a physiological position. The fiberglass gutter was fixed to the neck using a cohesive bandage for durable retention of synthetic splints (Haftelast, Lohmann and Rauscher, Neuwied, Germany) (Figure 4).

Due to the high plasticity of tissues in a several-day-old foal, the veterinary surgeon made a decision regarding conservative treatment and the closed repositioning of the dislocation under general injection anesthesia. The foal was premedicated with xylazine 0.5 mg/kg b.w. i.v. (Xylapan 20 mg/mL, Vetoquinol Biowet, Gorzów Wielkopolski, Poland) and flunixine meglumine 1.1 mg/kg b.w. i.v. (Vetaflunix 50 mg/mL, VET-AGRO, Lublin, Poland) and injection anesthesia was induced with diazepam 0.05 mg/kg b.w. i.v. (Solupam 5 mg/mL, Dechra, Northwich, UK). The foal was positioned in left lateral recymbency and correction of the C3 subluxation was performed manually. With the neck in maximum extension, both hands were placed on the transverse processes on each side of C3. Using a maximum force, the C3 was pushed ventrally and a series of latero-lateral radiographs were taken until a position was found in which all cervical vertebrae were in a physiological alignment. While maintaining this head and neck position, a fiberglass semicircular gutter was created to stabilize the neck in the desired position (Figure 3).

**Figure 5:**
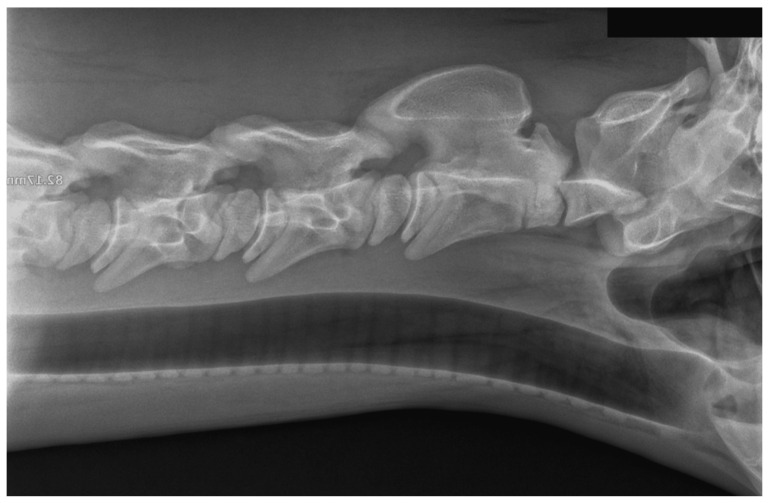
Radiograph of the neck in the latero-lateral projection. The radiograph was taken immediately after the removal of the external neck fixator (16 days after the manipulation).

The anesthesia and assisted recovery was completed without any complications. The foal could move freely and suckle from the mare in the applied stabilizer without any problems. The ataxia began to improve around day 12 post manipulation, and the fiberglass stabilizer was removed after 16 days post manipulation. After removing the external fixator, radiographs were taken to confirm the correct positioning of the cervical vertebrae (Figure 5).

**Figure 6:**
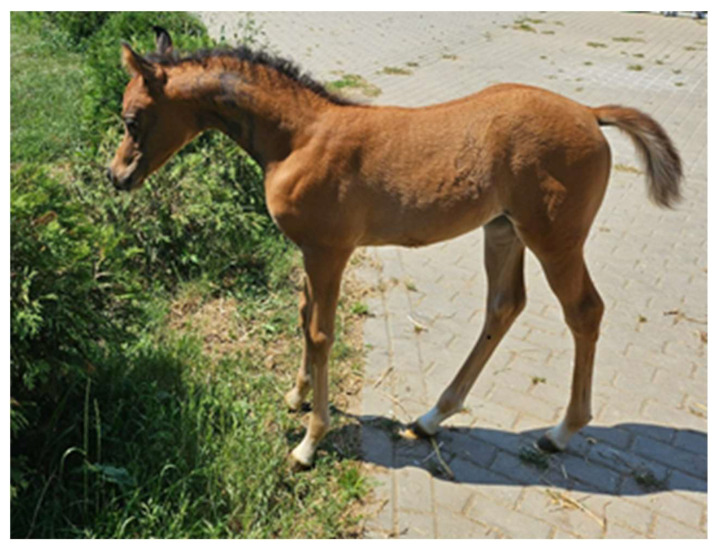
Foal with correct neck position after treatment.

The C2/C3 subluxation was no longer visible radiologically. After removing the fixator, the foal could assume a physiological neck posture (Figure 6).

After a week of observation in the hospital, the foal was discharged home. Follow-up videos from the owner were obtained 2 months after clinic discharge showing the foal moving freely without any neurological deficits.

## 3. Discussion

The described case of closed reduction in a cervical spine subluxation is relatively rare, and, with appropriate management, it can be treated with good results. Dislocations or fractures of vertebrae are an uncommon condition in horses, but the most common type of spine injury is damage to the cervical spine [1]. Foals usually suffer from injuries in the cervical spine. These can include subluxations, luxations, fractures of the cervical vertebrae and separation of the growth plates within the cervical vertebrae [3]. Subluxation can cause local compression of the spinal cord. In the further course of the disease, depending on the stability and position of the head and neck, permanent compression of the spinal cord can occur. Spinal cord compression can result from direct vertebrae damage or a post-traumatic hematoma within the spinal cord canal [2]. Depending on the location, extent and degree, luxation or subluxation results in varying degrees of spinal cord damage [1,6,7]. Clinical signs include abnormal neck flexion, hyperextension of the neck, tenderness on palpation and diffuse swelling of the injured area, mild or severe motor incoordination, recumbency, and even sudden death [1,8]. The factors that have the most significant impact on the clinical signs are the location and degree of spinal cord compression, which are of great importance for the degree of ataxia and, thus, the prognosis [9,10]. In untreated, chronic cases, due to the instability, degenerative joint disease develops, characterized by varying degrees of pain and neurological symptoms [11,12]. The etiology of cervical spine injuries is most often traumatic. These may include collisions with hard objects with a flexed neck, kicks, hyperflexion or excessive hyperextension of the neck during a fall after tripping, as well as collisions with another horse in a paddock [6,7]. In order to localize the site of spinal cord compression and determine whether it was caused by a fracture or displacement of the vertebra, various imaging techniques can be used, such as radiography, myelography, computed tomography or scintigraphy [8]. Fractures or subluxations of the cervical vertebrae can be treated both surgically and conservatively. The choice of treatment method depends on the degree of movement incoordination, stability of the fragments at the site of injury, and prognosis. In cases of severe discomfort and inability to move freely and safely, the animal should be euthanized. In acute post-traumatic cases, dexamethasone, non-steroidal anti-inflammatory drugs and/or DMSO are primarily used to reduce the swelling inside the spinal canal and limit the inflammatory reaction. A manual vertebral reposition is only possible in the case of relatively early-stage injuries with no permanent scarring of the surrounding tissues. Manual reposition was performed as Licka described [13]. However, as the author herself noted, the lack of head and neck stabilization after the procedure led to re-subluxation and had a fatal outcome as a consequence. In a similar case described a few years later by the same author, an external neck fixator was used which allowed the re-subluxation and its negative effects to be avoided [11]. Therefore, repositioning should be performed under X-ray control, and, after its completion, appropriate temporary external stabilization by a synthetic cast should be applied. In the absence of stabilization, re-subluxation or complete dislocation may occur, which may result in worsening neurological symptoms and even death [13]. In the case of foals, performing manual repositioning is relatively simple. Compared to older horses, it does not require extra external force, where an additional source of force may be necessary using an electrically powered hand pallet truck, as described by Gerlach [14]. If clinical signs do not improve despite the implementation of the described treatment, or scarring is observed in the surrounding soft tissues, surgical treatment options should be considered [15]. The goal of surgical therapy is to stabilize the lumen of the vertebral canal, which can be achieved by performing an internal fixation and intervertebral body fusion. However, challenges that remain with surgical vertebral interbody fusion surgeries include the invasiveness, costs and the lack of ability to predict the degree of improvement of neurological status, as well as future horse use [15]. In cases of long-lasting, severe motor ataxia, the prognosis is poor regardless of the method of therapy [1,16].

## 4. Conclusions

Manual repositioning of the cervical vertebrae in a case of subluxation is a less invasive alternative when surgical stabilization is not required. Stabilizing the foal’s neck in the repositioned posture with a ventral fiberglass stabilizer has effectively healed subluxation within the cervical spine.

## Figures and Tables

**Figure 4:**
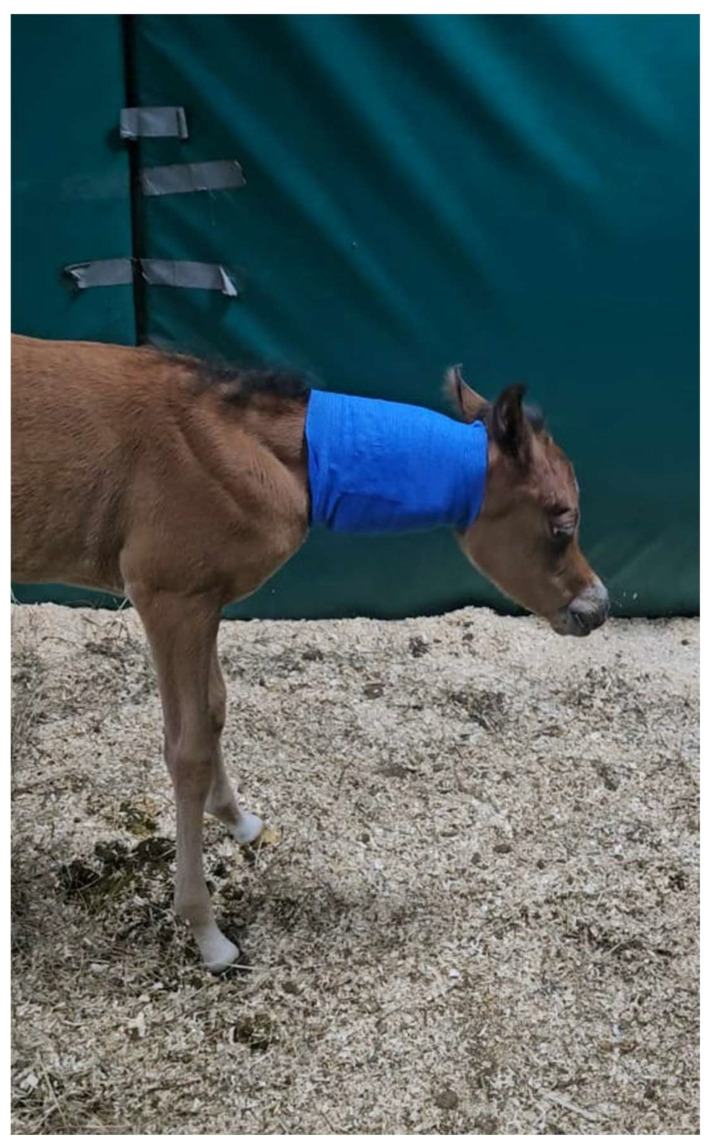
Foal after waking up from anesthesia following manual neck repositioning.

## Data Availability

All data analyzed during this study are included in this published manuscript.

## References

[1] Pinchbeck G., Murphy D. (2001). Cervical Vertebral Fracture in Three Foals. Equine Vet. Educ..

[2] Krunajevic T., Bergsten G. (1968). Luxation of The Cervical Spinal Column as a Cause of Wobbles in a Foal. Acta Vet. Scand..

[3] Cordula Gather T., Weinberger T., Nolting B. (2000). Halswirbelfraktur Bei Einem Pferd—Fallbericht. Pferdeheilkund.

[4] Barnes H.K., Crosby K., Talbot A., Baldwin C.M. (2023). Atlanto-Occipital Subluxation in an Adult Thoroughbred Gelding. Equine Vet. Educ..

[5] Watson A.G., Mayhew I.G. (1986). Familial Congenital Occipitoatlantoaxial Malformation (OAAM) in the Arabian Horse. Spine.

[6] Nixon A.J. (1996). Fractures of the Vertebrae. Equine Fracture Repair.

[7] Robertson J.L., Samii V., Auer J.A., Stick J.A., Kümmerle J.M., Prange T. (2005). Traumatic Disorder of the Spinal Column. Equine Surgery.

[8] Dyson S.J., Ross M.W., Dyson S.J. (2010). The Cervical Spine and Soft Tissue of the Neck. Diagnosis and Management of Lameness in the Horse.

[9] Tyler C.M., Davis R.E., Begg A.P., Hodgson D.R. (1993). A Survey of Neurological Diseases in Horses. Aust. Vet. J..

[10] Domańska-Kruppa N., Wierzbicka M., Stefanik E. (2024). Advances in the Clinical Diagnostics to Equine Back Pain: A Review of Imaging and Functional Modalities. Animals.

[11] Licka T. (2002). Closed Reduction of an Atlanto-Occipital and Atlantoaxial Dislocation in a Foal. Vet. Rec..

[12] Puangthong C., Bootcha R., Petchdee S., Chanda M. (2020). Chronic Atlantoaxial Luxation Imaging Features in a Pony with Intermittent Neck Stiffness. J. Equine Vet. Sci..

[13] Licka T., Edinger H. (2000). Temporary Successful Closed Reduction of an Atlantoaxial Luxation in a Horse from the Clinic for Orthopaedics in Ungulates. Vet. Comp. Orthop. Traumatol..

[14] Gerlach L., Muggli L., Lempe J., Breuer W., Brehm W. (2012). Successful closed reduction of an atlantoaxial luxation in a mature Warmblood horse. Equine Vet. Educ..

[15] Pezzanite L.M., Easley J.T., Bayless R., Aldrich E., Nelson B.B., Seim H.B., Nout-Lomas Y.S. (2022). Outcomes after Cervical Vertebral Interbody Fusion Using an Interbody Fusion Device and Polyaxial Pedicle Screw and Rod Construct in 10 Horses (2015–2019). Equine Vet. J..

[16] Reardon R., Kummer M., Lischer C. (2009). Ventral Locking Compression Plate for Treatment of Cervical Stenotic Myelopathy in a 3-Month-Old Warmblood Foal. Vet. Surg..

